# Microfluidic-Based Cationic Cholesterol Lipid siRNA Delivery Nanosystem: Highly Efficient In Vitro Gene Silencing and the Intracellular Behavior

**DOI:** 10.3390/ijms23073999

**Published:** 2022-04-03

**Authors:** Zhaoyuan Zhu, Li Zhang, Ruilong Sheng, Jian Chen

**Affiliations:** 1 School of Pharmacy, Shanghai Jiao Tong University, No. 800, Dongchuan Road, Shanghai 200240, China; zzygracephar0703@sjtu.edu.cn; 2 Instrumental Analysis Center, Shanghai Jiao Tong University, No. 800, Dongchuan Road, Shanghai 200240, China; lizhang09@sjtu.edu.cn; 3 CQM-Centro de Quimica da Madeira, Universidade da Madeira, Campus da Penteada, 9000-390 Funchal, Madeira, Portugal

**Keywords:** microfluidics, cationic cholesterol lipid, siRNA delivery, endocytosis pathway, gene silencing

## Abstract

Safe and efficient delivery of small interfering RNA (siRNA) is essential to gene therapy towards intervention of genetic diseases. Herein, we developed a novel cationic cholesterol lipid derivative (CEL) in which cholesterol hydrophobic skeleton was connected to L-lysine cationic headgroup via a hexanediol linker as the non-viral siRNA delivery carrier. Well-organized CEL/siRNA nanocomplexes (100–200 nm) were prepared by microfluidic-assisted assembly of CEL and siRNA at various N/P ratios. The CEL and CEL/siRNA nanocomplexes have lower cytotoxicity compared with bPEI25k. Delightfully, we disclosed that, in Hela–Luc and H1299–Luc cell lines, the micro-fluidic-based CEL/siRNA nanocomplexes exhibited high siRNA transfection efficiency under both serum-free condition (74–98%) and low-serum circumstances (80–87%), higher than that of lipofectamine 2000. These nanocomplexes also showed high cellular uptake through the caveolae/lipid-raft mediated endocytosis pathway, which may greatly contribute to transfection efficiency. Moreover, the time-dependent (0–12 h) dynamic intracellular imaging demonstrated the efficient delivery to cytoplasm after lysosomal co-localization. The results indicated that the microfluidic-based CEL/siRNA nanosystems possessed good stability, low cytotoxicity, high siRNA delivery efficiency, rapid cellular uptake and caveolae/lipid raft-dependent internalization. Additionally, this study provides a simple approach for preparing and applying a “helper lipid-free” cationic lipid siRNA delivery system as potential nanotherapeutics towards gene silencing treatment of (tumor) diseases.

## 1. Introduction

Construction of a gene delivery system with good biocompatibility, efficient gene loading and transfection efficiency, good payload stability and good scaling-up productivity has been regarded as one of the hot topics in current gene therapy [1]. Inspired by successful applications of non-viral vectors in safe and efficient gene delivery [2,3], in recent years, there has been a rapid development of non-viral vectors including cationic lipids and cationic polymers as promising nanocarriers for gene delivery [4]. For the sake of high performance and safe gene delivery, developing natural lipid-based gene carriers with good sustainability and increased value is highly desired, along with systematically exploring their related biological behaviors, including: cytotoxicity and biocompatibility, gene transfection efficiency and endocytosis mechanisms, as well as intracellular localization and trafficking [5].

Cholesterol, one of the essential natural lipids, is often involved in many important biological processes, such as membrane formation, lipid transport, metabolism, cell signal transduction and so on [6,7]. Cholesterol is also a commonly used steroid lipid for the construction of functional gene carriers [8]. The hydroxyl group endows cholesterol with chemical modifiability. Up to now, a couple of cationic cholesterol derivatives/lipids with high water solubility, certain positive charge and good molecular flexibility [9] were developed as promising gene carriers, in which cholesterol moiety serves as hydrophobic block and cationic headgroups (cationic polymers, cationic peptides, cationic amino acids and so on) act as gene (pDNA, small interfering RNA (siRNA) and microRNA) binding sites. The cholesterol moiety and cationic headgroups were connected together with functional linkers. Previous studies showed a couple of cholesterol-derived cationic lipids were utilized as highly efficient gene carriers. Zhi et al. reported that CHO-PGEA (cholesterol-terminated ethanolamine-aminated poly(glycidyl methacrylate)) efficiently delivered miR-182-in to suppress cardiac hypertrophy without organ injury [10]. The lipophilic cholesterol groups enhanced transfection efficiency while the hydrophilic hydroxyl groups endow high biocompatibility. Remant et al. disclosed that cholesterol grafting with low molecular weight polyethyleneimine (PEI, 0.6 and 1.2 kDa) enabled efficient silencing of the endogenous BCR-Ablonco gene in K562 cells [11]. Ehexige et al. developed the cationic peptidomimetic functionalized cholesterol through a robust chemical strategy to deliver siRNA against polo-like kinase 1 [12]. Lee et al. found that mono arginine-cholesterol with a cleavable ester bond could bind siRNA to form lipid nanoparticles, which exhibited a high level of target gene knockdown in vitro and in vivo for RNAi-based cancer therapy [13]. Therefore, it is feasible and valuable to further study cationic cholesterol derivatives as biomaterials for RNAi/siRNA delivery. According to our previous study, Sheng et al. confirmed a natural L-lysine cationic cholesterol derivative (Cho-es-Lys, CEL) to have high pGL3 luciferase DNA transfection efficiency, which almost reached the “gold standard” transfection level of branched-polyethyleneimine (bPEI)-25k [14]. However, its role in siRNA delivery remains unknown. Therefore, it is still of great significance to carry out in-depth study on siRNA delivery behaviors (such as nanocomplex stability, biocompatibility and gene silencing efficiency) of the cationic cholesterol derivative on the basis of good pDNA transfection capability. Besides, for many cationic cholesterol derivatives, the endocytosis pathway and intracellular trafficking/localization behavior are not fully clarified [15].

Studies have verified that intracellular behavior, transfection efficiency, cytotoxicity and other biological effects of gene-loaded nanosystems largely depend on the physico-chemical properties, such as nanoparticle size, morphology and surface charge [16,17]. Therefore, it is essential to precisely control the preparation process of gene-loaded nanosystems, particularly the electrostatic force-based self-assembly of cationic lipid/siRNA nanocomplexes, to achieve controllable nanosystems with desired physico-chemical properties [18]. To satisfy the demand, microfluidics, a powerful technology with high efficiency of mixing/self-assembling, has been widely used in the preparation of gene loaded nanoparticles. It shows superior reproducibility, better process control, automation and scale-up productivity with reduced time and simplified preparation procedures [19]. With the bloom of siRNA/ microRNA therapy, especially mRNA vaccine-based immuno-gene-therapy, microfluidics was employed as a promising and potential future technology for the manufacture of gene-loaded nanosystems [20,21]. Li et al. prepared siRNA/PAMAM polyamide nanocomplexes with different particle sizes and different geometric structures by microfluidic technology [16]. In producing lipid nanoparticles, Roces et al. evaluated operating and formulation parameters and Erica et al. further connected them with gene transfection behavior [22,23]. Kenichi et al. prepared core-shell nanoparticles through a novel way of two-step microfluidic-based mixing method [24]. In summary, microfluidic technology has broad prospects in lab-on-chip design and parameter adjustment as well as nanoformulation optimization [25,26]. However, in spite of research on polymer and lipid-based systems, to our knowledge, there is no report for a microfluidic-based cationic cholesterol derivatives/siRNA gene delivery nanosystem [27]. In addition, reproducible and stable preparation of siRNA-loaded nanomedicine by microfluidic technique guarantees more accurate results towards “precision siRNA gene therapy”.

Based on the above background and our previous study, herein, the cationic cholesterol derivative (Chol-es-Lys, CEL) was self-assembled with siRNA to prepare CEL/siRNA nanocomplexes with good reproducibly and high stability by microfluidic technology, and the average particle size, surface potential and morphology were characterized. The binding affinity of CEL–siRNA and the stability of CEL/siRNA nanocomplexes were evaluated by agarose gel retardation. The cytotoxicity of CEL and nanocomplexes was measured by Cell Counting Kit (CCK)-8 assay with half maximal inhibitory concentration (IC_50_). The gene transfection (gene silencing) efficiency of anti-luciferase siRNA, which can effectively knock down the firefly luciferase gene, was evaluated by luciferase assay in HeLa–Luc and H1299–Luc cell lines. To explore the mechanism, we measured the cell uptake efficiency, analyzed the endocytosis pathway and observed the dynamic intracellular localization/trafficking of CEL/siRNA nanocomplexes. The results showed that with the help of microfluidic technology, ideal CEL/siRNA nanocomplexes were prepared at a proper charge ratio between amine groups of CEL and phosphate groups of siRNA (N/P ratio). The CEL/siRNA nanocomplexes showed superior gene delivery efficiency, low cytotoxicity and rapid internalization by cells, based on good stability and high serum-resistance. This study provides a comprehensive and convincing study of cationic cholesterol derivative CEL on siRNA delivery towards gene silencing treatment of (tumor) diseases.

## 2. Results and Discussion

### 2.1. Characterization of the Cationic Cholesterol Derivative and Nanocomplexes

Firstly, cationic cholesterol derivative (Chol-es-Lys, CEL) was successfully synthesized (isolated yield: 62%) by connecting the cholesterol hydrophobic skeleton with natural cationic L-lysine headgroup through a hexanediol linker with carbonate/ester bonds, respectively. The structure of CEL was characterized by the ^1^H nuclear magnetic resonance (NMR) spectrum (Figure S2). The amino group of L-lysine showed two proton peaks at 7.78 and 8.42 ppm, respectively. The obvious peaks at 1.00 and 0.67 ppm were the methyl peaks on the steroidal ring and another three side-chain methyl peaks located between 0.85 and 0.92 ppm. The signal of methylene linked to the oxygen atom (-CH_2_OCO-) at around 4.10 ppm confirmed the successful linkage by hexanediol.

**Figure 1:**
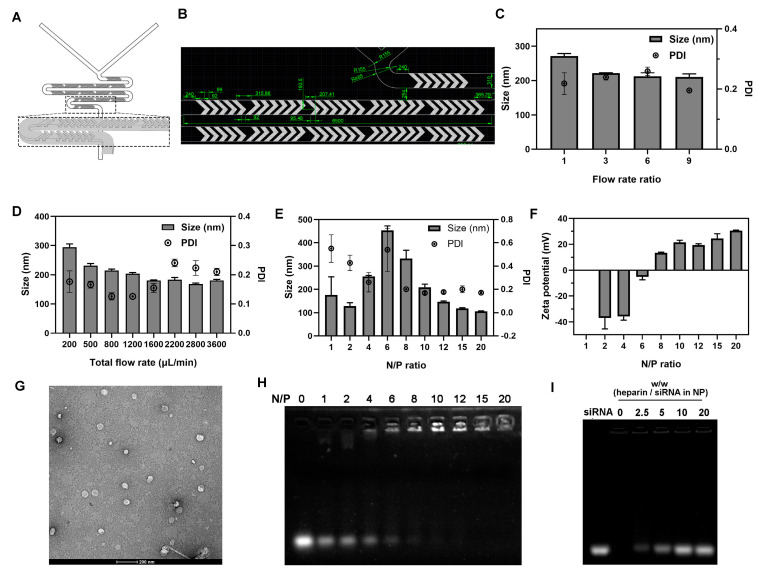
Characterization of Chol-es-Lys/siRNA nanocomplexes. (**A**) The structure and (**B**) the microscopic size of the staggered herringbone micromixer chip. The optimization process of (**C**) flow rate ratio and (**D**) total flow rate as microfluidics parameters. (**E**) Particle size and (**F**) surface charge of microfluidic-based Chol-es-Lys/siRNA nanocomplexes at different N/P ratios. (**G**) Morphology of Chol-es-Lys/siRNA nanocomplexes (N/P = 15) by transmission electron microscope. (**H**) siRNA binding affinity for Chol-es-Lys under various N/P ratio. (**I**) Heparin displacement assay of Chol-es-Lys/siRNA nanocomplexes at N/P = 15. PDI, polydispersity index; NP, referred to Chol-es-Lys/siRNA nanocomplexes.

The cationic L-lysine headgroup of Chol-es-Lys could bind/load siRNA via electrostatic force (positive–negative charge interaction) in aqueous phase, so as to form nanocomplexes. To prepare well-organized nanocomplexes, the binding/mixing process of Chol-es-Lys and siRNA was controlled by microfluidic technology. Previous studies have shown that the hydrodynamic particle size, morphology and surface charge of nanocomplexes greatly affected gene transfection and intercellular transport [28,29,30]. Herein, the hydrodynamic particle size and surface charge of CEL/siRNA nanocomplexes under various N/P ratios were measured by dynamic light scattering (DLS) instrument. First of all, we screened the optimal flow rate ratio (FRR) and total flow rate (TFR) by Figure 1C,D. Figure 1C illustrated that the particle size reached minimum at FRR = 9 with favorable polydispersity index (PDI). Then, this study chose TFR = 1200 μL/min after weighing the particle size and PDI (Figure 1D). It can offer the best PDI to guarantee the quality of nanocomplexes, even though the particle size was not the smallest. As a result, the following microfluidic-based preparation of CEL/siRNA nanocomplexes was carried out at FRR = 9 and TFR = 1200 μL/min. As shown in Figure 1E,F, CEL/siRNA nanocomplexes showed different average particle sizes and surface charges under different N/P ratios with microfluidic conditions. The particle sizes of nanocomplexes were about 100–200 nm at N/P ratios of 10–20, which were decreased with the increase in N/P ratio with controllable low PDI value (<0.2) and were stable under each N/P condition. Notably, under the control of microfluidic technology, the nanocomplexes prepared in different batches had relatively homogenous nanoparticle sizes and low PDI, which provides a strong basis for scale-up production and application of the nanocomplexes in future study. Meanwhile, the surface charges of CEL/siRNA nanocomplexes increased from –40 mV to 30 mV, with N/P ratios increasing from 2 to 20. Moreover, the transmission electron microscope (TEM) image showed that CEL/siRNA nanocomplexes were nearly spherical-shaped nanoparticles with the diameters of –50 nm (Figure 1G). The small particle size in the TEM image compared to the DLS result was attributed to the shrinkage of nanocomplexes during the air-drying process. It could be noticed that the appropriate molecular flexibility and protonation capability of CEL endowed efficient and well-organized nanoparticle formation [31]; the “soft and flexible” hydrophobic tail in the cholesterol allows CEL to interact with siRNA to form condensed and small-sized nanocomplexes. The positive surface charge is largely related to the protonation capability of the L-lysine headgroup (isoelectric point: pI = 9.8), the 20–30 mV positive charges of the nanocomplexes could benefit their adsorption/adhesion to negatively-charged cell membranes, thus causing efficient cellular uptake.

### 2.2. Interaction between CEL and siRNA

The binding capacity of cationic cholesterol derivative CEL to siRNA was evaluated by agarose gel electrophoresis. siRNA binding/condensation experiment (Figure 1H) showed that, in tris-borate-EDTA (TBE) buffer solution, the brightness of free-siRNA migration bands gradually decreased with the increasing amount of CEL, and the siRNA was completely retarded at N/P = 8. Previous studies have demonstrated that the binding affinity between cholesterol-based cationic lipids and siRNA largely depends on the pKa value of the cationic headgroup [32].

To evaluate the binding stability of CEL to siRNA, heparin displacement assay was conducted with agarose gel electrophoresis (Figure 1I). Negatively-charged heparin (pKa = 3.1) could induce dissociation of lipid/siRNA nanocomplexes by competing binding with cationic lipids. After addition of increased amounts of heparin (weight ratio: *w*/*w* = 2.5–20), siRNA migration bands with enhanced brightness gradually appeared from the loading wells of nanocomplexes, indicating the heparin-induced dissociation of the nanocomplex. According to the literature reports, the flexible and hydrophobic tail cholesterol can enhance the stability of their siRNA nanocomplexes [33].

### 2.3. Stability of CEL/siRNA Nanocomplexes

To meet the requirement of in vivo application, siRNA-loaded nanocomplexes need to have high stability to avoid the dissociation and maintain a good protection effect of siRNA against enzyme digestion/protein binding under physiological conditions before reaching the target cells/tissues [34].

**Figure 2:**
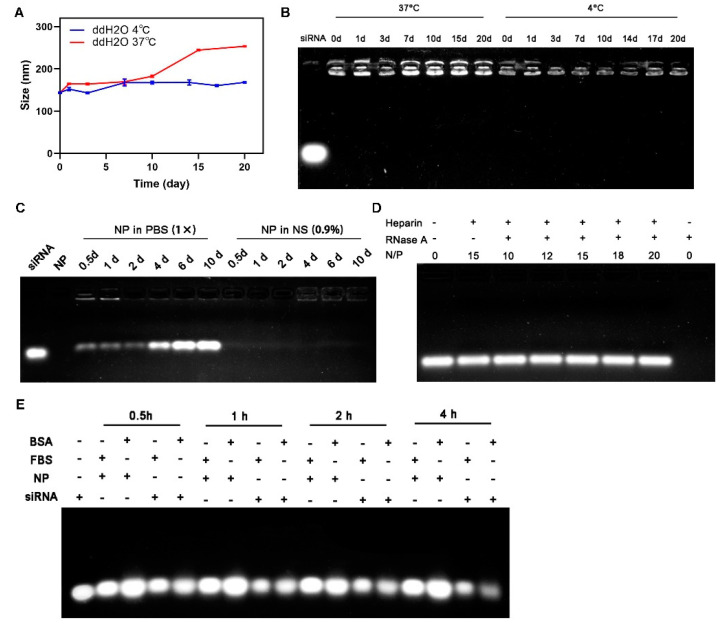
Stability of Chol-es-Lys/siRNA nanocomplexes. Storage stability of Chol-es-Lys/siRNA nanocomplexes (N/P = 15) evaluated by (**A**) average sizes and (**B**) agarose gel electrophoresis. (**C**) Stability of Chol-es-Lys/siRNA nanocomplexes (N/P = 15) in salt solutions. (**D**) RNase protection assay of Chol-es-Lys/siRNA nanocomplexes (N/P = 10, 12, 15, 18, 20). (**E**) Serum stability assay of Chol-es-Lys/siRNA nanocomplexes (N/P = 15) in 10% FBS or 10% BSA. NS, normal saline; FBS, fetal bovine serum; BSA, bull serum albumin; NP, referred to Chol-es-Lys/siRNA nanocomplexes.

First, time-dependent hydrodynamic particle size of CEL/siRNA nanocomplexes (N/P = 15) was monitored by DLS; it showed that the nanocomplexes could maintain particle size stability for about 20 days under low-temperature (4 °C) storage (Figure 2A). Under physiological temperature conditions (37 °C), the particle size of the nanocomplexes was basically stable within 10 days and slowly increased about 70–90 nm after 10 days. Agarose gel electrophoresis showed that CEL and siRNA could maintain good binding stability at both low temperature and physiological temperature conditions (Figure 2B), indicating that, during long-term storage, the electrostatic force-based CEL and siRNA binding affinity was not affected by storage time and environmental temperature, and the loaded-siRNA would not spontaneously run out of nanocomplexes. Moreover, the CEL/siRNA nanocomplexes (N/P = 15) have high stability in a normal isotonic saline (NS) environment, however, in isotonic PBS with high salt concentration, the CEL/siRNA nanocomplexes (N/P = 15) showed lower stability, and the loaded-siRNA would be slowly released from the nanocomplexes after 0.5 days (Figure 2C).

The RNase A degradation assay confirmed that CEL/siRNA nanocomplexes could efficiently protect the loaded-siRNA against enzyme degradation. After incubation in RNase A, the integrity of siRNA was assessed by gel electrophoresis (Figure 2D). Bright migration bands of siRNA were observed without tailing or diffusion, indicating the CEL/siRNA nanocomplexes under the N/P ratios of 10, 12, 15, 18 and 20 were well-protected against RNase A. By contrast, the free/naked-siRNA (without CEL protection) was completely degraded (no bright band observed) under RNase A. Serum stability assay further confirmed that the CEL/siRNA nanocomplexes (N/P = 15) could well-protect siRNA against serum protein interference (Figure 2E). After incubation with 10% fetal bovine serum (FBS) or 10% bull serum albumin (BSA) for 0–4 h, no obvious brightness change in the siRNA band was observed on CEL/siRNA nanocomplexes, suggesting their high serum protein-resistance capability, while the brightness of the naked siRNA band significantly decreased with longer incubation time. In addition, the hydrodynamic sizes of the particles in the media of 10% FBS were shown in Supplementary Table S1. The above results demonstrated that CEL/siRNA nanocomplexes could maintain high stability under simulated physiological conditions and exhibit excellent protective effect against RNase A and serum proteins.

### 2.4. Cytotoxicity of the Cationic Cholesterol Derivative and Nanocomplex by CCK-8 Assay

**Figure 3:**
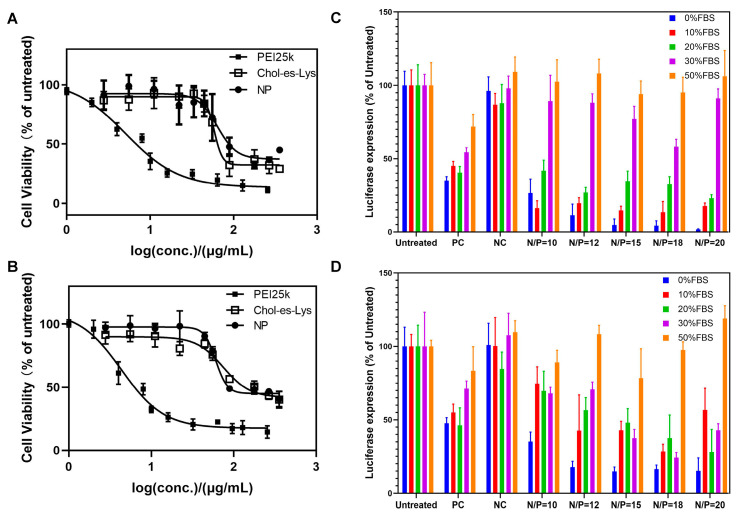
Transfection safety and performance of the cationic cholesterol derivative Chol-es-Lys and Chol-es-Lys/siRNA nanocomplexes. Cytotoxicity was evaluated with various concentrations (0–350 µg/mL) in (**A**) H1299–Luc cells and (**B**) HeLa–Luc cells. The groups treated with no materials or nanocomplexes were set as 100% cell viability. Luciferase gene silencing efficiencies of Chol-es-Lys/siRNA nanocomplexes (N/P = 10, 12, 15, 18, 20) with different serum concentrations in (**C**) H1299–Luc cells and (**D**) HeLa–Luc cells with siRNA concentration at 100 nM. PC, lipofectamine 2000 /siRNA in 100 nM as positive control; NC, Chol-es-Lys/NC-siRNA nanocomplexes as negative control. NP, referred to Chol-es-Lys/siRNA nanocomplexes; conc., concentration; PEI, polyethyleneimine; FBS, fetal bovine serum.

Low cytotoxicity and high biocompatibility were considered as the pre-requisite of cationic gene vectors towards safe gene delivery application. The in vitro cytotoxicity of CEL and its siRNA-loaded nanocomplexes in H1299–Luc and HeLa–Luc cells was evaluated by CCK-8 assay using commercially available bPEI-25k as a control. As shown in Figure 3A,B, it can be observed that bPEI-25k showed high cytotoxicity (IC_50_ = 5.10 µg/mL in H1299–Luc cells and IC_50_ = 4.36 µg/mL in HeLa–Luc cells), which is possibly due to the cell membrane destruction effect of the L-lysine cationic headgroup. On the contrary, the inhibitory effects of CEL and its preparation on cell proliferation were much lower, and the IC_50_ was about 10 times higher than that of bPEI-25k. CEL showed relatively acceptable cytotoxicity (IC_50_ = 58.57 µg/mL in H1299–Luc cells and IC_50_ = 74.59 µg/mL in HeLa–Luc cells) in the concentration range of 0–350 μM. These cells still showed strong proliferation activity, especially in the low concentration range (<55 µg/mL). The nanocomplexes also demonstrated low cytotoxicity (IC_50_ = 63.69 µg/mL in H1299–Luc cells and IC_50_ = 60.50 µg/mL in HeLa–Luc cells) and no significant inhibition on cell proliferation activity at the concentration up to 55 µg/mL, which was much higher than the actual dosage of gene transfection. The results confirmed that the cationic lipid CEL has high biocompatibility. Previous studies have shown that the cytotoxicity of the cationic cholesterol lipid gene carriers largely depends on their cationic moieties and/or headgroups [35]. The natural L-lysine cationic headgroup designed for CEL has less cytotoxicity, which makes it a potential safe gene carrier for siRNA gene silencing treatment. Although CEL was only verified in tumor cells, it could be anticipated that they may also possess low cytotoxicity in normal human cells, which could increase their applicability in the cancer gene therapy under the co-existence of normal human cell lines.

### 2.5. Luciferase Gene Silencing Efficiencies of CEL/siRNA Nanocomplexes in the Absence/Presence of Serum

The luciferase gene silencing efficiency of CEL was evaluated in H1299–Luc and HeLa–Luc cells in the absence/presence of serum, so as to investigate the dependence from the serum concentration and N/P ratio. Accordingly, CEL/siRNA nanocomplexes were prepared by aforementioned microfluidic technology under different N/P ratios. The luciferase expression of the cell groups without any siRNA treatment was set as 100%, and the commercially available lipofectamine 2000 (lipo2000) was utilized as the positive control. The cells were transfected by CEL/siRNA nanocomplexes under serum-free, low serum (10%, 20%) and high serum (30%, 50%) conditions, respectively. The siRNA concentration was 100 nM. As shown in Figure 3C, under serum-free conditions, CEL/siRNA nanocomplexes showed extremely high luciferase gene silencing ability in H1299–Luc cells, which was enhanced along with the increasing of N/P ratio (74–98%) and was even (15–30 times) higher than the lipo2000 control group. Under the condition of low serum, its gene silencing effect was slightly weakened, but still better than that of lipo2000 (80–87% vs. 55% in 10% serum; 60–77% vs. 60% in 20% serum). The nanocomplexes with N/P = 10 showed the most obvious weakening at 20% serum concentration. It may be due to the electrostatic interactions between a large number of negatively-charged serum proteins and positively-charged nanocomplexes, which weakened the siRNA binding affinity and thus diminished the nanocomplexes’ stability. However, with the increase in serum concentration to 30%, the nanocomplexes almost lost 80% of their transfection (gene silencing) capability. Especially, the low N/P nanocomplexes (N/P = 10 and 12) almost lost 90% of their transfection capability and the gene silencing effect of middle N/P nanocomplexes (N/P = 15 and 18) were no better than that of lipo2000 (23–42% vs. 46%); at 50% serum, the nanocomplexes almost have no gene silencing capability. Similar results were also found in the gene silencing profiles of HeLa–Luc cells (Figure 3D). Compared to lipo2000, the siRNA transfection performance of CEL/siRNA nanocomplexes at low serum concentration exhibited less advantage, but the nanocomplexes still maintained their leading position in 30% serum environment. Mikhail et al. showed that some cationic cholesterol derivatives have 80% silencing effect in serum-free conditions, while only 20% under 10% serum [35]. Widchaya et al. compared the pDNA delivery effects of several cationic cholesterol derivatives with various cationic headgroups and linkers [31], they disclosed that L-lysine headgroup-containing lipids showed the same gene delivery performance as lipo2000 at serum-free or low serum level. Remant et al. utilized low molecular weight PEI as the cationic headgroup of cholesterol derivatives, showing a 55% gene silencing effect, but less than that of PEI25k [11]. In comparison to the previous results, the cationic cholesterol derivative CEL in this study exhibited outstanding gene silencing efficiency under serum-free and low serum environments. It is well known that negatively charged serum proteins could interfere in the siRNA binding/loading affinity of cationic lipid nanoparticles, thus many gene delivery studies evade in-serum transfection. The encouraging gene silencing results in the presence of low serum suggested that cationic cholesterol lipid CEL, to some extent, could condense and load siRNA into nanocomplexes regardless of proteins and therefore lead to efficient in-serum siRNA delivery. In addition, within the siRNA transfection dose range (≤100 nM, N/P = 10–20), CEL and CEL/siRNA nanocomplexes both showed low cytotoxicity (>90% cell viability), further confirming that CEL could be used as a relatively safe, efficient and promising lipid siRNA carrier towards practical gene silencing application.

### 2.6. Cellular Uptake Capability of CEL/Cy5-siRNA Nanocomplexes

**Figure 4:**
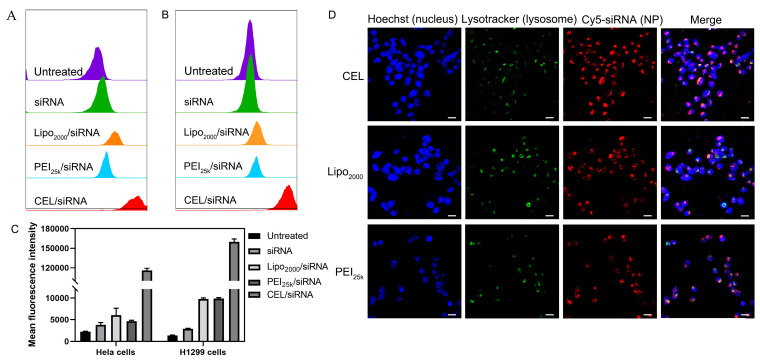
Cell uptake performance of Chol-es-Lys/Cy5-siRNA nanocomplex. Cellular uptake efficiency of Chol-es-Lys/Cy5-siRNA nanocomplexes analyzed by flow cytometry in (**A**) H1299–Luc cells and (**B**) HeLa–Luc cells. The cell groups without nanocomplexes were set as Untreated. (**C**) Mean fluorescence intensities recorded in assay of panel (**A**,**B**). (**D**) Cell uptake in the HeLa–Luc cells observed by confocal laser scanning microscopy (scale bar = 25 µm). Line 1: CEL/Cy5-siRNA nanocomplexes; Line 2: Lipo2000/Cy5-siRNA nanocomplexes; Line 3: PEI/Cy5-siRNA nanocomplexes. CEL, Chol-es-Lys. PEI, polyethyleneimine.

Cell uptake behavior, specifically manifested as the uptake amount and proportion of nanocomplexes, is closely related to the related gene transfection efficiency. Herein we carried out a cell uptake assay by fluorescence activated cell sorting (FACS) of the CEL/Cy5-siRNA nanocomplexes, in which Cy5 labeled siRNA was used as a fluorescence index to analyze the uptake efficiency of nanocomplexes by H1299–Luc cells and HeLa–Luc cells. The uptake capability was quantitatively expressed by the mean fluorescence intensity (MFI); high MFI value means higher uptake amount/ratio of nanocomplexes that entered the cells. FACS data showed the highest fluorescence-positive signals in H1299–Luc cells after treatment with CEL/Cy5-siRNA nanocomplexes for 4 h, while lipo2000/Cy5-siRNA and PEI25k /Cy5-siRNA treatment groups also showed much weaker fluorescence-positive signals compared to those of CEL/Cy5-siRNA nanocomplexes (Figure 4A). Similar results were also presented in HeLa–Luc cells (Figure 4B). It could be seen that in HeLa–Luc cells, the MFI of the CEL/Cy5-siRNA nanocomplexes’ treatment group was about 13.5 times that of the lipo2000/Cy5-siRNA treatment group and 18 times that of the PEI25k/Cy5-siRNA treatment group, respectively (Figure 4C). The results suggested that CEL/Cy5-siRNA nanocomplexes had high cell uptake efficiency, which meant that they were easily internalized into the HeLa–Luc cells within a short time. The high cellular uptake of the nanocomplexes may bring them high gene transfection efficiency [36,37]. Under low serum condition (10% FBS), CEL/Cy5-siRNA nanocomplexes possess high cellular uptake efficiency, while high serum environment with high concentration of serum-proteins/enzymes would hinder cell uptake behavior and reduced the amount of nanocomplexes being “swallowed” (internalized) by the cells. In addition, confocal laser scanning microscopy (CLSM) images illustrated that CEL, lipo2000 and PEI25k could effectively transfect siRNA into HeLa–Luc cells (Figure 4D). Under the same treatment time, the fluorescence intensity of CEL/Cy5-siRNA nanocomplexes was higher than that of lipo2000/Cy5-siRNA or PEI25k/Cy5-siRNA, which was consistent with the results of FACS in HeLa Luc cells. It could be noticed that, to some extent, the remarkable gene transfection efficiency of CEL/Cy5-siRNA nanocomplexes maybe be due to their high uptake capability/efficiency by the cells. Moreover, it is necessary to further study their endocytosis mechanism to provide more information for understanding the related cell uptake process.

### 2.7. Endocytosis Pathway Analysis of CEL/Cy5-siRNA Nanocomplexes

**Figure 5:**
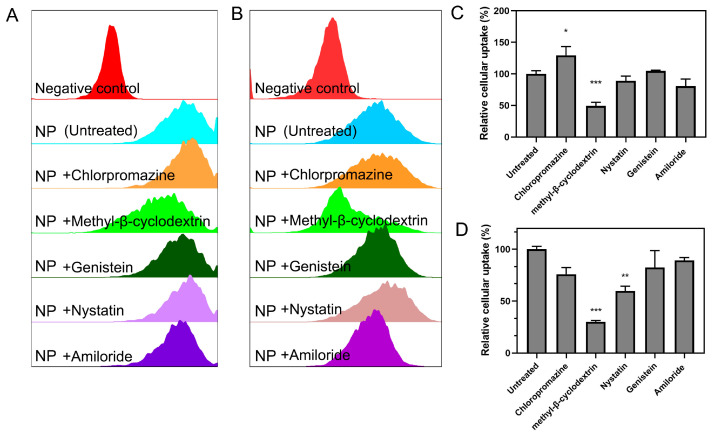
Endocytosis pathway analysis of CEL/Cy5-siRNA nanocomplexes. Endocytosis pathway analysis of CEL/Cy5-siRNA nanocomplexes in (**A**) H1299–Luc cells and (**B**) HeLa–Luc cells by flow cytometry in the presence of endocytosis-specific inhibitors. Either nanocomplexes nor endocytosis-specific inhibitors were applied to the negative control group. NP refers to Chol-es-Lys/Cy5-siRNA nanocomplexes. The cells fed with nanocomplexes but no inhibitors were also referred to as the untreated group. Relative cellular uptake in (**C**) H1299–Luc cells and (**D**) HeLa–Luc cells calculated with mean fluorescence intensities in assay of panel (**A**,**B**) with the untreated group as the 100%. Clathrin-mediated endocytosis inhibitor: chloropromazine. Caveolae-mediated endocytosis: methyl-β-cyclodextrin; Genistein, Nystatin. Macropinocytosis mediated endocytosis inhibitor: Amiloride (***p < 0.001, **p < 0.01, *p < 0.05).

Based on the high siRNA transfection efficiency and prominent cell uptake efficiency of CEL/Cy5-siRNA nanocomplexes, we further focused on their intracellular transport behavior and explored the main endocytosis pathways affecting cell uptake. In a pre-experiment of cholesterol gateway inhibition, we disclosed that the endocytosis pathway was generally cholesterol-dependent. Herein, we conducted the endocytosis pathway analysis of the CEL/Cy5-siRNA nanocomplexes with several specific endocytosis inhibitors. As shown in Figure 5A, the relative cell uptake of CEL/Cy5-siRNA nanocomplexes decreased drastically in both cells (50% in H1299–Luc cells and 70% in HeLa–Luc cells) in the presence of methyl-β-cyclodextrin (Mβ-CD, an inhibitor of caveolae/lipid raft-mediated endocytosis pathway). Caveolae, a special type of lipid raft, is rich in cholesterol and glycosphingolipids. Mβ-CD can extract cholesterol from caveolae on the cytoplasmic membrane by forming a water-soluble inclusion complex, causing the disintegration of caveolae and lipid raft and, therefore, blocked the cellular uptake process of CEL/Cy5-siRNA nanocomplexes. It suggested that the cationic cholesterol-containing gene carriers could deliver siRNAs into cells mainly through the caveolae/lipid raft-mediated endocytosis pathway. Moreover, the addition of Nystatin (another caveolae/lipid raft-mediated endocytosis inhibitor) reduced the relative cell uptake rate to about 60% in HeLa–Luc cells, while slightly declined that in H1299–Luc cells. It can be explained that hydro-Nystatin binds to cholesterol (in caveolae/lipid raft) by hydrophobic chain interaction, and the Mβ-CD binds to cholesterol (in caveolae/lipid raft) via inclusive interaction, which made Nystatin and Mβ-CD demonstrate different binding capability to cholesterol [38,39]. However, the presence of Amiloride (macropinocytosis inhibitor) and Genistein (tyrosine kinase, caveolae and phagocytosis inhibitor) had no significant effect on the relative uptake rate of cells. Interestingly, the relative uptake rate of H1299–Luc cells slightly increased under the action of chlorpromazine (clathrin-mediated endocytosis inhibitor), which may be attributed to the protonation effect of chlorpromazine that promoted the activity of CEL/siRNA nanocomplexes to some extent. The results indicated that, CEL/siRNA nanocomplexes could be effectively internalized by H1299–Luc cells and HeLa–Luc cells mainly through the caveolae/lipid raft mediated pathway, which is widely considered to be the cholesterol-dependent endocytosis process.

As it is well known that the endocytosis mechanism usually affects gene transfection efficiency by altering the intracellular distribution of gene-loaded nanocomplexes. Previous studies revealed that clathrin-mediated endocytosis and micropinocytosis led to a “staggering exocytosis” and adverse “lysosome entrapment” effect to siRNA delivery, which encouraged an alteration of the intracellular trafficking pathway to bypass lysosomes [40,41]. However, it is not so clear which pathway should be the best choice of an effective endocytosis process during siRNA transfection. Since the complexity of physical–chemical nanocomplexes and diversity of transfected cell types, the “preferred endocytosis pathway” remains controversial. Recent research disclosed that the caveolae-mediated endocytosis route could allow gene/drug-loaded nanoparticles to bypass the lysosome and smoothly reach the cytoplasm, e.g., Song et al. found that caveolae-mediated endocytosis was conductive to the robust siRNA delivery of mannose-modified trimethyl chitosan-cysteine/tripolyphosphate nanoparticles through the subcellular organelle of the Golgi-complex and endoplasmic reticulum [42]. Qiu et al. designed endoplasmic reticulum (ER) membrane-decorated siRNA nanoparticles to effectively transport siRNA through the endosome–Golgi–ER pathway to avoid lysosomal degradation [43]. In this study, caveolae/lipid raft-mediated pathways dominated the cell uptake mechanism of CEL/siRNA nanocomplexes, which inferred that high transfection efficiency was attributed to the cholesterol-dependent caveolae/lipid raft mediated pathways. Thus, it exhibited that caveolae-mediated endocytosis may possess the greatest siRNA delivery potential. It may not only assist CEL/siRNA nanocomplexes in circumventing the endosome–lysosome pathway, but also facilitate siRNA release on the endoplasmic reticulum when targeting mRNA behaviors. Moreover, the study on CEL/siRNA nanocomplexes provided an approach for improving the efficiency of siRNA delivery from the selection of the endocytosis pathway by utilizing cholesterol-containing gene delivery nanosystem.

### 2.8. Intracellular Localization and Trafficking of CEL/Cy5-siRNA Nanocomplexes

**Figure 6:**
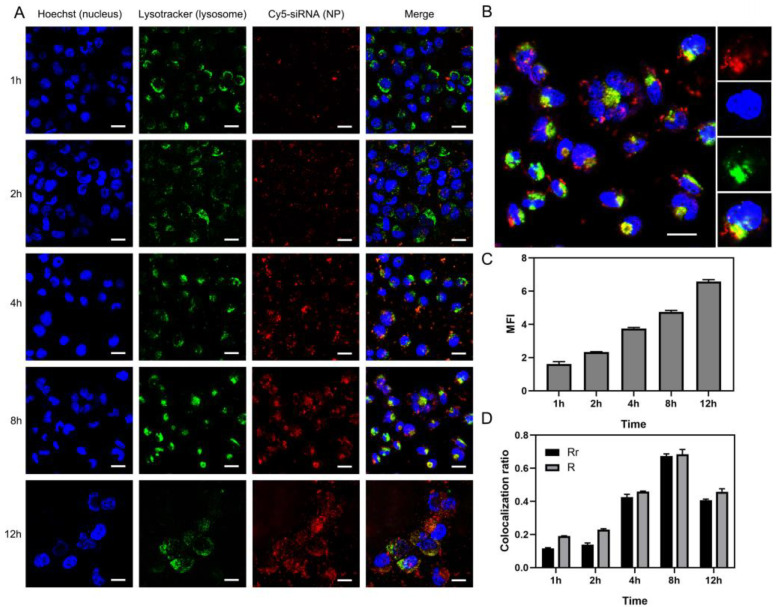
Subcellular localization and lysosomal escaping of CEL/Cy5-siRNA nanocomplexes in the HeLa–Luc cells. (**A**) Subcellular localization of CEL/Cy5-siRNA nanocomplexes in the HeLa–Luc cells observed with confocal laser scanning microscopy at different transfection time points. Nuclei were stained with Hoechst (blue), lysosomes were stained by Lysotracker (green). Cy5-siRNA (red) presented the location of nanocomplexes. (**B**) Colocalization of CEL/Cy5-siRNA nanocomplexes (red) with lysosomes (green) in the HeLa–Luc cells at 8 h. A large amount of yellow fluorescence in the cells was observed, which was the result of the overlap between green fluorescence and red fluorescence. (**C**) MFIs of CEL/Cy5-siRNA nanocomplexes at different time points. (**D**) Lysosomal colocalization ratios of Cy5-siRNA and Lysotracker green-stained lysosomes calculated from multiple images (n = 3). Rr, Pearson correlation coefficient; R, Mander’s overlap coefficient. The colocalization ratios and MFIs were analyzed with Image J software. scale bar = 25 µm. CEL, Chol-es-Lys, MFI, mean fluorescence intensity.

Furthermore, intracellular localization and behavior of the internalized CEL/Cy5-siRNA nanocomplexes were observed by confocal microscopy. Previous studies discovered that some specific intracellular trafficking pathways, such as endosome–lysosome–cytoplasm pathway and endosome–Golgi–ER–cytoplasm pathway, have been involved during siRNA delivery [43]. As far as siRNA transfection was concerned, the former pathway demands lysosomal capture and escape, and the latter represents a more effective cytoplasmic delivery. In our study, with the extension of internalization time, it could be seen that the intracellular red fluorescence intensity gradually increased (Figure 6A,C), indicating that CEL/Cy5-siRNA nanocomplexes were continuously taken up and internalized into the cells. The confocal image was captured and the colocalization finder of Image J software was used to analyze the co-localization between green fluorescence and red fluorescence. The results showed that the non-significant intracellular co-localization was observed before 4 h; their Pearson correlation coefficient (RR) and Mander’s overlap coefficient (R) were relatively low, while the co-localization phenomenon started to emerge between 4 h and 8 h, as the yellow fluorescence obtained by the superposition of red and green in the merge column was obviously increased, and RR and R approached the peak at 8 h (Figure 6B). At 12 h, the co-localization was weakened, with RR and R decreased, in addition to the dimmed green lysosomal fluorescence (Figure 6A,D). The above results demonstrated that CEL/Cy5-siRNA nanocomplexes could be continuously internalized by the cells and thus releasing the loaded siRNA into the cells. It is speculated that, in the early stage, CEL/Cy5-siRNA nanocomplexes were internalized by cells, most of which were transported to the cytoplasm through other organelles to facilitate mRNA degradation, suggesting that high transfection and uptake could be achieved with only 4 h of treatment in the above experiments. The results were in accordance with the cellular uptake studies, while some of the CEL/Cy5-siRNA nanocomplexes were captured and escaped in lysosomes in the middle-late stage. This has been observed by the weakening of lysosomal fluorescence as well as the colocalization phenomenon and coefficient, which was in accordance with our previous co-localization results [33]. Some studies have shown that free cholesterol can enhance endosomal escape, since it can promote the fusion of lipid nanoparticles and endosomal membrane by stabilizing the local double-layer curvature [44]. Furthermore, the strong interaction between cationic L-lysine and anionic lysosomal membrane could also induce the disruption of lysosome. It was speculated that CEL/Cy5-siRNA nanocomplexes could diminish the membrane stability through cholesterol–lipid bilayer hydrophobic and electrostatic interactions, so as to realize the “lysosomal escape” effect and thus increase the gene silencing efficiency.

## 3. Materials and Methods

### 3.1. Materials

Cholesteryl chloroformate (98.0%), 1,6-hexanediol (99.0%), L-2,6-bis ((tert- butoxycarbonyl)amino) hexanoic acid (Nα-,Nε-bis-Boc-L-lysine, 98.0%), Genistein (98.0%) and Amiloride (99.0%) were provided by Bidepharm. The anti-luciferase siRNA duplex sequences were synthesized by Ruibo Bio company (Guangzhou, China; sequence: 5′-GCGCUGCUGGUGCCAACCCTT-3′, 5′-GGGUUGGCACCAGCAGCAGCGCTT-3′), as well as the non-specific and disordered NC-siRNA as negative control. HEPES, bull serum albumin, TBE buffer and RNase A solution were purchased from Sangon Biotech (Shanghai, China). RNA loading buffer was purchased from Takara Bio (Dalian, China), GelRed nucleic acid staining dye from Biotium (San Francisco Bay Area, CA, USA) and agarose from Yeasen (Shanghai, China). Branched PEI with a molecular weight of 25 kDa (bPEI25) was provided by Sigma-Aldrich (St. Louis, MO, USA), and the Lipofectamine^®^ 2000, LysoTracker Green DND-26 was obtained from Invitrogen life technologies (Carlsbad, CA, USA). The reagents for cell culture were all purchased from ThermoFisher Scientific (Carlsbad, CA, USA), including dulbecco′s modified eagle medium (DMEM), FBS, BSA, trypsin and penicillin-streptomycin, in addition to BCA assay kit. HeLa–Luc cells and H1299–Luc cells (a cervical cancer cell line and a liver cancer cell line both stably expressing firefly luciferase) were obtained from our laboratory. Cell Counting Kit-8, Hoechst 33342 was purchased in Beyotime Biotechnology (Shanghai, China). Cell lysis buffer and luciferase assay reagent were procured from Promega Corporation (Madison, WI, USA). Chloropromazine (98.0%), Mβ-CD (99.0%) and Nystatin (98.0%) were the products of Macklin (Shanghai, China).

### 3.2. Synthesis Routes and NMR Spectra of the Cationic Cholesterol Derivative CEL

The synthesis steps of cationic cholesterol derivatives (Chol-es-Lys, CEL) and their important intermediates were described in detail in Supplementary Material Figure S1, according to our previous study [14,45]. The NMR spectra of CEL (dissolved in CDCl_3_) were characterized on a 400 MHz Fourier transform NMR spectrometer (BRUKER AVANCE III, Zurich, Switzerland) at ambient temperature (Supplementary Material Figure S2).

### 3.3. Preparation and Characterization of Chol-es-Lys/siRNA Nanocomplexes

Preparation of CEL/siRNA nanocomplexes was carried out in two-inlet staggered herringbone micromixer (SHM) chips by microfluidics. The structure was illustrated in Figure 1A,B, clearly showing the microscopic size and shape characteristic. siRNA lyophilized powder was diluted with diethylpyrocarbonate (DEPC)-treated ddH_2_O to 2 μM and the solution was injected from one inlet, while CEL was dissolved with DEPC-treated ddH_2_O to different concentrations according to N/P ratio and injected from another inlet using syringe pumps (Langer, LSP01-2A and LSP02-1B, Hebei, China), similar to our previous study [46]. Initially, the single factor experiment was designed to optimize two important parameters, FRR and TFR. Optimal FRR was chosen from the range of 1:1, 3:1, 6:1 and 9:1 according to the particle sizes and PDI, where 9:1 means the flow rate of siRNA solution was 9 while the flow rate of CEL solution was 1, respectively [47]. During the process, other factors were controlled as TFR = 800 μL/min and N/P ratio = 10. Following that, the optimal TFR was selected from the range of 200–3600 μL/min with the optimal FRR and N/P ratio = 10. After the optimization above, the TFR was set as 1.2 mL/min, and the FRR of siRNA:CEL as 9:1. To prepare the CEL/siRNA nanocomplexes, the impact of the N/P ratio from 1 to 20 was investigated in this study.

The particle size, PDI and zeta potential of the prepared nanocomplexes were measured by DLS on a Zeta-sizer Nano ZS-90 (Malvern Instruments, Malvern, UK). Briefly, a 0.5 mL sample was added into micro pools and measured directly after preparation without any dilution. All samples were prepared in triplicate and, for each sample, three measurements were conducted. The morphologies of these nanoparticles were visualized by a transmission electron microscope (Talos L120C G2, Thermo-Fisher, Carlsbad, CA, USA) under 120 kV acceleration voltage equipped with a CCD camera.

### 3.4. Formation and Dissociation Analysis of CEL/siRNA Nanocomplexes

The interaction between CEL and siRNA was evaluated on the above microfluidic-based CEL/siRNA nanocomplexes (N/P ratio from 0 to 20) by an agarose gel retardation assay. To evaluate the siRNA binding affinity, the nanocomplexes were added into 6× loading buffer and loaded onto a 1% (*w/v*) agarose gel containing 1‰ GelRed. Electrophoresis was performed at 110V for 20 min in TBE buffer on a BioRad Mini-PROTEAN Petra system (Hercules, CA, USA), and the siRNA bands were viewed under ultraviolet irradiation and photographed using an automatic gel image analysis system (Tanon-2500B, Shanghai, China). In order to further study the binding stability of CEL and siRNA, a heparin displacement assay was performed by pre-incubating the nanocomplexes with sodium heparin in different weight ratios to siRNA (0, 2.5, 5.0, 10.0, 20.0) for 1 h at 37 °C. The resultant nanocomplex solutions were electrophoresed on a 1% agarose gel, as described above.

### 3.5. Stability of CEL/siRNA Nanocomplexes

Storage stability was firstly assessed by DLS and agarose gel electrophoresis. The prepared CEL/siRNA nanocomplexes (N/P = 15) were kept at 4 °C and 37 °C for 20 days, and samples at different time intervals were collected to monitor size distribution and binding stability. The detailed methods were similar to those described in Experimental Section 2.3 and Section 2.4. Similarly, the stability of CEL/siRNA nanocomplexes (N/P = 15) in the solution was tested in PBS (10 mM) and normal saline (0.9% *w/w*) for 10 days, respectively. The binding situation was visualized by agarose gel electrophoresis.

RNase protection assay was carried out to assess the protection ability of CEL to siRNA in form of nanocomplexes using agarose gel electrophoresis [48]. CEL/siRNA nanocomplexes (N/P = 10, 12, 15, 18, 20) were incubated with 1 µL RNase A at 37 °C for 2 h. A positive control (siRNA without RNase A treated) and a negative control (siRNA treated with RNase A) were included. Following incubation, the samples were fully mixed with EDTA (200 mM) to inactivate the RNase A and added with heparin (1:40 *w*/*w*) to dissociate siRNA from the nanocomplexes. After that, the samples were electrophoresed on a 1% agarose gel, as described above.

A serum stability assay was performed according to previously reported procedures [32]. The prepared CEL/siRNA nanocomplexes (N/P = 15) were mixed with equal volumes of 20% FBS or BSA (pH 7.4), respectively, and incubated at 37 °C for 0.5, 1, 2 and 4 h. Free siRNA without serum/protein treated was a positive control. At each time point, samples were water-bathed at 60 °C for 5 min to terminate serum activity. Following that, the samples were incubated with heparin (1:40 *w/w*) for 1 h to replace siRNA from the nanocomplex. The siRNA bands were visualized by agarose gel electrophoresis with the automatic gel image analysis system, as described above.

### 3.6. Cytotoxicity of CEL and CEL/siRNA Nanocomplexes by CCK-8 Assay

Cytotoxicity of the cationic cholesterol derivative CEL and CEL/siRNA nanocomplexes was evaluated in H1299–Luc and HeLa–Luc cells by CCK-8 assay. Cells were firstly seeded into 96-well microplates at 8 × 10^3^ cells per well, and were cultivated under 37 °C and 5% CO_2_ for 24 h in 100 µL DMEM medium (with 10% FBS). Subsequently, fresh DMEM medium (with 10% FBS) took replacement, and the cationic cholesterol derivative and nanocomplexes under a series of concentrations were individually and respectively added into the wells (each sample with six replicates (n = 6)). After a further incubation for 48 h, CCK-8 detection reagent was added and co-incubated for 1.5 h according to the guidance, and an enzyme labeling instrument (Tecan infinite M200 Pro, Männedorf, Switzerland) was used for detecting the ultraviolet absorption at 450 nm. Commercially available bPEI-25k was utilized as a reference. The wells, untreated, were set as 100% cell viability and the curves were drawn as cell viability-log (concentration) to calculate the IC50.

### 3.7. Luciferase Gene Silencing Efficiencies of CEL/siRNA Nanocomplexes in the Absence/Presence of Serum

HeLa–Luc cells and H1299–Luc cells, both stably expressing firefly luciferase, were seeded at a density of 3 × 10^5^ cells per well in 48-well plates, and incubated at 37 °C and 5% CO_2_ with DMEM (10% FBS) overnight. Then, the as-prepared CEL/siRNA nanocomplex/lipoplex (N/P = 10, 12, 15, 18 and 20) solutions were diluted in 200 µL FBS-free DMEM medium or DMEM with 10%, 20%, 30% and 50% FBS and incubated for 30 min. The nanocomplexes containing anti-luciferase siRNA were used in the experimental group while those composed of the non-specific and disordered NC-siRNA were used as the negative control. Moreover, the commercially available lipo2000 loaded with anti-luciferase siRNA was used as a positive control. The siRNA concentration was set as 100 nM according to the protocol of the siRNA synthesis company and previous experiment results (Figure S3). The CEL concentrations were 21, 25.2, 31.5, 37.8 and 42 μM, corresponding to 10, 12, 15, 18, and 20 N/P ratios. Then, the nanocomplex solutions were added into the plates and the cells were incubated for 4 h in 0%, 10%, 20%, 30% and 50% FBS-containing DMEM. The medium was replaced with 200 µL fresh DMEM medium (with 10% FBS) and further incubated for 44 h. Each sample was in five replicates (n = 5), and three measurements were conducted.

Following that, the luciferase transfection assays were conducted in accordance with the protocol of the Promega Luciferase assay system. Briefly, the medium was discarded and cells were washed twice with PBS. Cell lysis buffer (80 µL) was then added to each well, and the plate was gently shaken for 30 min. The lysates were centrifuged at 4000×*g* for 5 min to deposit cell debris in a microplate centrifuge. Thereafter, 50 µL of the supernatant was transferred to tubes, followed with the addition of 50 µL of luciferase assay reagent. Luminescence was read on an illuminometer (Berthold, Bad Wildbad, Germany) at the absorbance wavelength of 562 nm. Finally, total protein was determined by the BCA assay kit using 20 µL of cell lysate. Results were represented as relative light units (RLUs)/μg protein and converted to percentage luciferase expressed against the control.

### 3.8. Cellular Uptake of CEL/Cy5-siRNA Nanocomplexes

Cy5-labeled siRNA (Cy5-siRNA) was provided by labeling Cy5 on the 5-UtR of siRNA. The CEL/Cy5-siRNA nanocomplexes (N/P = 15) were obtained as per the methods described in Section 2.3, while the bPEI-25k/Cy5-siRNA (N/P = 10) and lipo2000/Cy5-siRNA were used as positive references.

HeLa–Luc and H1299–Luc cells were seeded into 24-well plates at a density of 5 × 10^4^ cells/well and cultivated in DMEM medium (with 10% FBS) for 24 h. The cells were subsequently treated with the CEL/Cy5-siRNA nanocomplexes under various N/P ratios and incubated for 4 h in serum-free DMEM medium. After that, the cells were washed with PBS for three times, digested by trypsinization, harvested by centrifugation and re-suspension in 1 mL PBS solution. Finally, each sample was transferred (in triplicate) to a flow cytometry (BD FACS Calibur, Franklin Lakes, NJ, USA). The cells were gated by sideward scatter versus forward scatter plots in FACS analysis. The Cy5 fluorescence intensities were recorded in the PE-Cy7-A channel and the mean fluorescence intensities (MFIs) were calculated.

A confocal laser scanning microscope (Leica TCS SP8, Leica, Wetzlar, Germany) was also used to visually show the cellular uptake after 4 h incubation. The detailed methods were described in Section 3.10 below.

### 3.9. Endocytosis Pathway Analysis of CEL/Cy5-siRNA Nanocomplexes

H1299–Luc and HeLa–Luc cells were seeded into 24-well plates at a density of 5 × 10^4^ cells/well, respectively, and incubated in DMEM medium (with 10% FBS) at 37 °C with 5% CO_2_ for 24 h. The endocytosis-related inhibitors, including chloropromazine (clathrin-mediated endocytosis pathway inhibitor, 10 µg/mL), Mβ-CD (caveolae-mediated pathway inhibitor, 10 mM), Genistein (caveolae-mediated endocytosis pathway inhibitor, 100 μM), Nystatin (caveolae-mediated endocytosis pathway inhibitor, 54 μM) and Amiloride (micropinocytosis pathway inhibitor, 100 μM), were separately added into the cells for 1 h pre-treatment [49,50,51,52]. Next, the medium was replaced with fresh serum-free medium, and the as-prepared CEL/Cy5-siRNA nanocomplexes (siRNA 75 nM, N/P = 15) were added and incubated for 4 h. The cells fed with nanocomplexes without inhibition were set as the positive control (the untreated group without inhibitor). The cells were harvested for analysis by flow cytometry as Section 2.8 illustrated. Relative cellular uptake in two cell lines were individually calculated from MFIs, for which MFIs in the untreated group were the 100%.

### 3.10. Intracellular Localization and Trafficking of CEL/Cy5-siRNA Nanocomplexes

Hoechst 33342, Lysotracker Green DND-26 and Cy5-siRNA were used to detect the subcellular localization and intracellular trafficking of CEL/Cy5-siRNA nanocomplexes in vitro. HeLa–Luc cells were seeded into cell slides in 24-well plates at a density of 5 × 10^4^ cells/well and incubated in DMEM medium (with 10% FBS) at 37 °C with 5% CO_2_ overnight until the cells adhered to the slides. The cells were transfected with the as-prepared CEL/Cy5–siRNA nanocomplexes (siRNA 75 nM, N/P = 15) by co-incubation up to 4 h. The subcellular localization and intracellular trafficking were monitored within 12 h (at time intervals of 2 h, 4 h, 8 h and 12 h). For time points at 8 h and 12 h, the medium was replaced by fresh medium with 10% FBS after 4 h incubation. The staining procedure was performed as the previous reference [33,53]. To be specific, at different time points, the treated cells were washed with PBS three times to remove residual nanocomplexes. Lysosomes were stained by incubating the cells with LysoTracker Green DND-26 (75 nM) at 37 °C for 25 min to specifically stain endosome/lysosome organelles, followed by three washes with PBS. The cell nuclei were stained with Hoechst 33342 (2 μg/mL) for 5 min at room temperature, followed by three washes with PBS. The living cells were visualized without any fixation, and the images were captured by a scanning confocal microscope (Leica TCS SP8, Leica, Wetzlar, Germany) using a 63× oil objective.

### 3.11. Statistical Analysis

Data were reported as mean ± standard deviation or percentage. Statistical analysis was performed by using GraphPad Prism Version 8 (GraphPad Software, San Diego, CA, USA) and SPSS Statistics 25 (IBM, Armonk, NY, USA). Independent t test was performed to compare between two groups, while one-way analysis of variance (ANOVA) was performed to analyze comparison of multiple groups. ANOVAs were corrected for multiple comparison by Tukey (Gaussian distribution) and Kruskal–Wallis (non-Gaussian distribution) tests. *p* > 0.05 was considered not significant.

## 4. Conclusions

In this study, we successfully developed cholesterol derivative CEL/siRNA nanocomplexes as an ideal siRNA delivery system with superior in vitro gene silencing effect. The microfluidic-based CEL/siRNA nanosystem has the advantages of simple, facile, easy-to-manipulate and good potential for scaling-up production. The nanocomplexes could efficiently load siRNA with good stability and protect siRNA against RNase A and serum protein. The cationic cholesterol derivative CEL and CEL/siRNA nanocomplexes exhibited low toxicity and significantly high gene silencing effect (under serum-free/low serum conditions) in H1299–Luc and HeLa–Luc cells; they could be internalized into the cells mainly through caveolae–lipid raft mediated pathways and undergo endosome–lysosome–cytoplasm intracellular trafficking pathways. In addition, this study provided an efficient approach for creating a natural lipid-derived, microfluidic-based, well-organized and low-toxic siRNA delivery nanosystem for high performance gene silencing towards gene therapy application.

## Data Availability

The data that support the findings of this study are available from the corresponding author upon reasonable request.

## Supplementary Materials

Supplementary Materials can be found at https://www.mdpi.com/article/10.3390/ijms23073999/s1. Reference [14] is cited in the supplementary materials.

## Abbreviations

siRNA	small interfering RNA
PEI	polyethyleneimine
IC50	half maximal inhibitory concentration
CEL	Chol-es-Lys
TBE	tris-borate-EDTA
FBS	fetal bovine serum
BSA	bull serum albumin
FACS	fluorescence activated cell sorting
Mβ-CD	methyl-β-cyclodextrin
MFI	mean fluorescence intensity
