# Supplementary material for: Microfluidic-Based Cationic Cholesterol Lipid siRNA Delivery Nanosystem: Highly Efficient In Vitro Gene Silencing and the Intracellular Behavior
Source: Int J Mol Sci. 2022 Apr 3;23(7):3999. doi: 10.3390/ijms23073999 (PMC8999516; doi:10.3390/ijms23073999)
Supplement: Supplementary file 1 [file ijms-23-03999-s001.zip › ijms-1640176-supplementary.pdf]

Supplementary Material of

Microfluidic-Based Cationic Cholesterol Lipid siRNA Delivery Nanosystem: Highly Efficient In Vitro Gene Silencing and the Intracellular Behavior

Zhaoyuan Zhu [1], Li Zhang [2], Ruilong Sheng [3,*] and Jian Chen [1,*]

1 School of Pharmacy, Shanghai Jiao Tong University, No.800, Dongchuan Road, Shanghai 200240, China; zzygracephar0703@sjtu.edu.cn
2 Instrumental Analysis Center, Shanghai Jiao Tong University, No.800, Dongchuan Road,
Shanghai 200240, China; lizhang09@sjtu.edu.cn
3 CQM-Centro de Quimica da Madeira, Universidade da Madeira, Campus da Penteada,
9000-390 Funchal, Madeira, Portugal
* Corresponding: ruilong.sheng@staff.uma.pt (R.S.); chenjian@sjtu.edu.cn (J.C.)

Figure S1. Synthesis routes and molecular structures of Cho-lys-es lipids

Figure S2. Nuclear magnetic resonance spectrum of Chol-es-Lys

Figure S3. Luciferase gene silencing efficiencies of CEL/siRNA nanocomplexes with different concentrations

Table S1. The hydrodynamic sizes of the particles in the media of 10% FBS

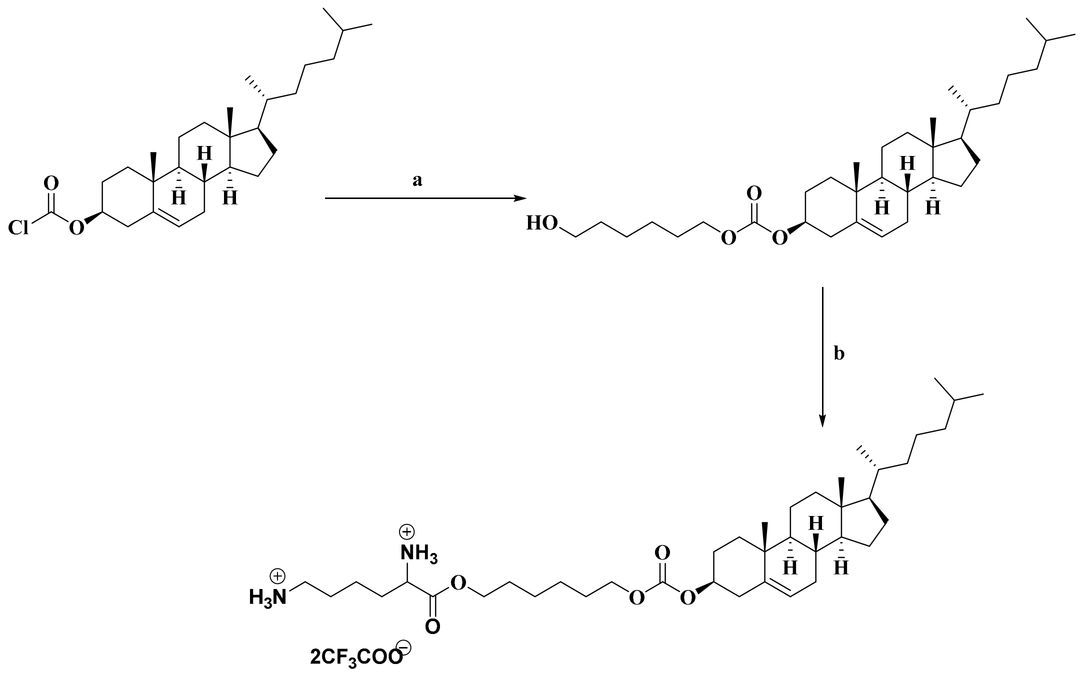

Figure S1. Synthesis routes and molecular structures of Cho-lys-es lipids

(a) 1,6-hexanediol, CH_2Cl_2/pyridine, 12h, r.t. (b) 1. Nα-,Nε-bis-Boc-L-lysine, DCC/DMAP, CH_2Cl_2, 24 h, r.t.; 2. trifluoroacetic acid, 0°C, CH_2Cl_2, 0.5h.

The synthesis of Cho-es-Lys lipid could be accomplished within several simple and facile steps.[1] First, cholesteryl chloroformate was reacted with excess of 1,6-hexanediol to prepare Cholest-5-en-3-yloxycarbonyloxyhexan-6-ol (Cho-es-OH) as a precursor in 74% yield, which was then acylated with L-2,6-bis((tert-butoxycarbonyl)amino) hexanoic acid (Nα-,Nε-bis-Boc-L-lysine). After that, the Boc groups were deprotected in TFA at 0°C to produce two carbon-ate ester linkage bearing cholesterol cationic lipids Cho-es-Lys (yield: 62%)

1.R. Sheng, T. Luo, H. Li, J. Sun, Z. Wang and A. Cao, *Colloids And Surfaces B-Biointerfaces*, **2014**, *116*, 32-40.

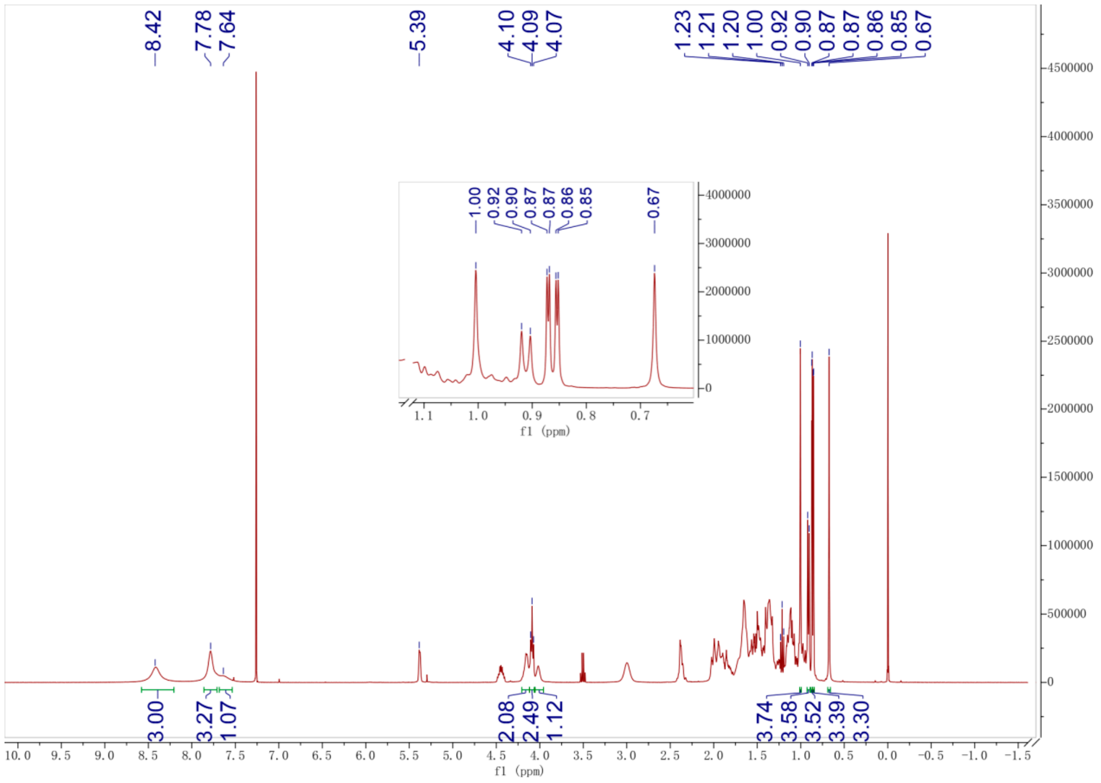

Figure S2. nuclear magnetic resonance spectrum of Chol-es-Lys

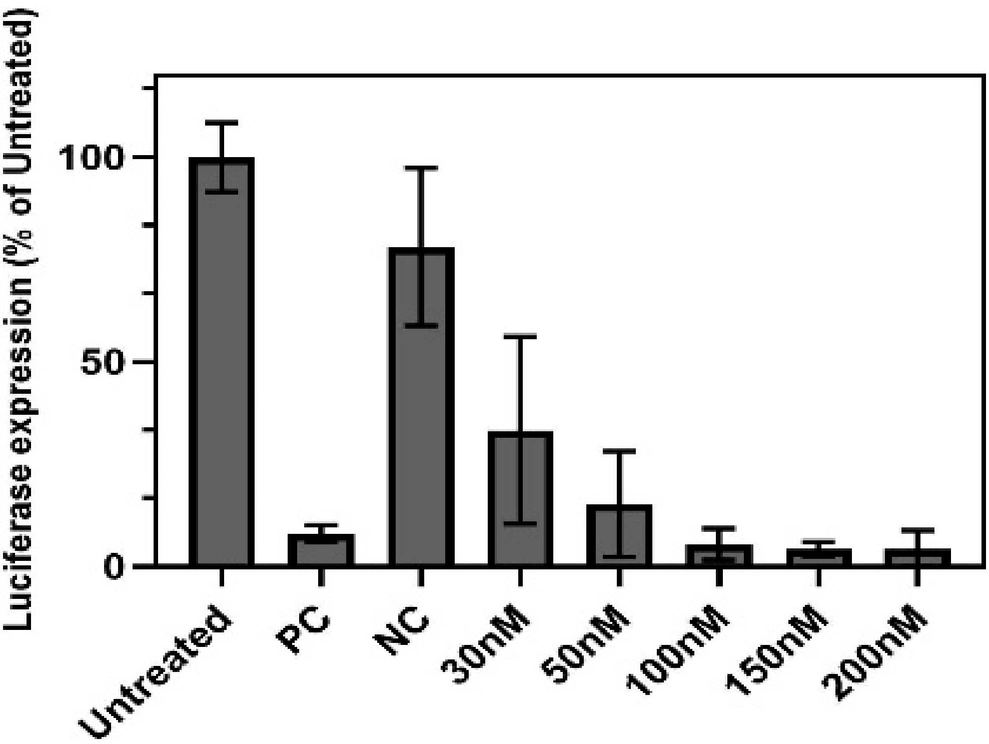

Figure S3. Luciferase gene silencing efficiencies of CEL/siRNA nanocomplexes with different concentrations

PC, lipofectamine 2000 as positive control in 100nM; NC, CEL/NC-siRNA nanocomplexes as negative control. CEL, Chol-es-Lys.

Table S1. The hydrodynamic sizes of the particles in the media of 10% FBS

Time	Hydrodynamic sizes of the particles (nm)	PDI
0h	125.4 ± 4.4	0.213
4h	129.8 ± 14.5	0.252
24h	375.0 ± 37.6	0.338
72h	812.8 ± 102.1	0.359
